# Utility of *ASNS* gene methylation evaluated with the HPLC method as a pharmacogenomic biomarker to predict asparaginase sensitivity in BCP-ALL

**DOI:** 10.1080/15592294.2023.2268814

**Published:** 2023-10-15

**Authors:** Atsushi Watanabe, Kunio Miyake, Yuriko Yamada, Ei-Ichiro Sunamura, Takuya Yotani, Keiko Kagami, Shin Kasai, Minori Tamai, Daisuke Harama, Koshi Akahane, Kumiko Goi, Kimiyoshi Sakaguchi, Hiroaki Goto, Shinichiro Kitahara, Takeshi Inukai

**Affiliations:** a Department of Pediatrics Environmental Medicine, School of Medicine, University of Yamanashi, Yamanashi, Japan; b Department of Epidemiology and Environmental Medicine, School of Medicine, University of Yamanashi, Yamanashi, Japan; c Tsukuba Research Institute, Research and Development, Sekisui Medical Co, Ltd, Ibaraki, Japan; d Instrument System Development Center, Research and Development, Sekisui Medical Co, Ltd, Ibaraki, Japan; e Department of Pediatrics, Hamamatsu University School of Medicine, Shizuoka, Japan; f Hematology/Oncology, Kanagawa Children’s Medical Center, Kanagawa, Japan; g R&D Management Department, Research and Development, Sekisui Medical Co, Ltd, Tokyo, Japan

**Keywords:** DNA methylation, acute lymphoblastic leukaemia, asparagine synthetase, high-performance liquid chromatography, precision medicine

## Abstract

Asparaginase is an important agent for the treatment of acute lymphoblastic leukaemia (ALL), but it is occasionally associated with severe adverse events. Thus, for safer and more efficacious therapy, a clinical biomarker predicting asparaginase sensitivity is highly anticipated. Asparaginase depletes serum asparagine by deaminating asparagine into aspartic acid, and ALL cells are thought to be sensitive to asparaginase due to reduced asparagine synthetase (ASNS) activity. We have recently shown that allele-specific methylation of the *ASNS* gene is highly involved in asparaginase sensitivity in B-precursor ALL (BCP-ALL) by using next-generation sequence (NGS) analysis of bisulphite PCR products of the genomic DNA. Here, we sought to confirm the utility of methylation status of the *ASNS* gene evaluated with high-performance liquid chromatography (HPLC) analysis of bisulphite PCR products for future clinical applications. In the global methylation status of 23 CpG sites at the boundary region of promoter and exon 1 of the *ASNS* gene, a strong positive correlation was confirmed between the mean percent methylation evaluated with the HPLC method and that with the NGS method in 79 BCP-ALL cell lines (R^2^ = 0.85, *p* = 1.3 × 10^−33^) and in 63 BCP-ALL clinical samples (R^2^ = 0.84, *p* = 5.0 × 10^−26^). Moreover, methylation status of the *ASNS* gene evaluated with the HPLC method was significantly associated with *in vitro* asparaginase sensitivities as well as gene and protein expression levels of ASNS. These observations indicated that the *ASNS* gene methylation status evaluated with the HPLC method is a reliable biomarker for predicting the asparaginase sensitivity of BCP-ALL.

## Introduction

DNA methylation is an epigenetic modification that leads to changes in gene expression. In cancer, aberrant methylation of CpG islands in the promoter region is involved in diverse biological and clinical features of cancer, including the treatment response. To improve therapeutic outcomes in cancer patients, a reliable analytic system to evaluate the methylation status of the specific genes involved in drug sensitivities of cancer cells is desirable. For the treatment of acute lymphoblastic leukaemia (ALL) and malignant lymphoma patients, asparaginase is an important agent [1]. The introduction [2,3] and intensification [1,4–7] of asparagine therapy dramatically improved therapeutic outcomes in childhood ALL. Of note, recent clinical observations of patients who could not tolerate asparaginase therapy due to adverse events having poorer outcomes [8–10] also clearly demonstrated the importance of asparaginase therapy in current treatment for childhood ALL. Asparaginase depletes serum asparagine by deaminating asparagine into aspartic acid. ALL cells are supposed to be sensitive to asparaginase due to reduced asparagine synthetase (ASNS) activity.

Despite its clinical importance, asparaginase therapy is occasionally associated with severe adverse events, including hypersensitivity, coagulopathy, and acute pancreatitis [11,12]. Thus, for safer and more efficacious asparaginase therapy, a clinical biomarker predicting asparaginase sensitivity is highly anticipated. As a clinical biomarker for asparaginase sensitivity, *in vitro* asparaginase sensitivities of primary samples could be the most straightforward biomarker. In the early studies, success rates of *in vitro* drug sensitivity assay were reportedly 61–80% [13,14], and fresh samples were required for a higher success rate [13]. In recent studies, to improve the success rate, mesenchymal stromal cells (MCSs) have been used as feeders [15]. However, MSCs reportedly protect ALL cells from asparaginase cytotoxicity *in vitro* by producing asparagine [16]. Under these circumstances, an alternative biomarker that is simple and evaluable even in frozen samples would be required in clinical practice. By using next-generation sequence (NGS) analysis of bisulphite PCR products of genomic DNA, we have recently shown that an allele-specific methylation of the *ASNS* gene due to aberrant genome imprinting is highly involved in asparaginase sensitivity and is associated with karyotypes in B-precursor ALL (BCP-ALL) [17] and T-ALL [18].

In the present study, we qualitatively and quantitatively evaluated the *ASNS* methylation status by high-performance liquid chromatography (HPLC) and confirmed its reliability and utility as a pharmacogenomic biomarker for asparaginase sensitivity of ALL cells.

## Methods

Genomic DNA was extracted from 79 BCP-ALL cell lines and 63 BCP-ALL clinical samples, as we reported previously in an earlier study [17]. The present study was approved by the ethics committee at the University of Yamanashi and Sekisui Medical Co. Ltd. Bisulphite modification of genomic DNA was performed using an EZ DNA Methylation Lightning Kit (Zymo Research, Irvine, CA). Amplification was performed with FastStart Taq DNA Polymerase (Roche, Basel, Switzerland). Polymerase chain reaction (PCR) primers for bisulphite PCR were designed using MethPrimer (https://www.urogene.org/methprimer/). The sequences of forward and reverse primers were 5’-AGTTTTGAGGTTATTTTTAAATAGG-3’ and 5’-AAAACCAAAAATATAAACAACTTAAC-3’, respectively. Precise methods for NGS analysis of bisulphite PCR products were previously reported [17]. NGS reads (with complete deciphering of 23 CpG sites) were applied for evaluation of methylation status. The heatmap with hierarchical clustering and the histogram of methylation statuses of each read were generated using gplots package ver.3.3.1 and ggplot2 package ver.3.3.5 of R ver.4.1.1 software.

**Figure 1.**
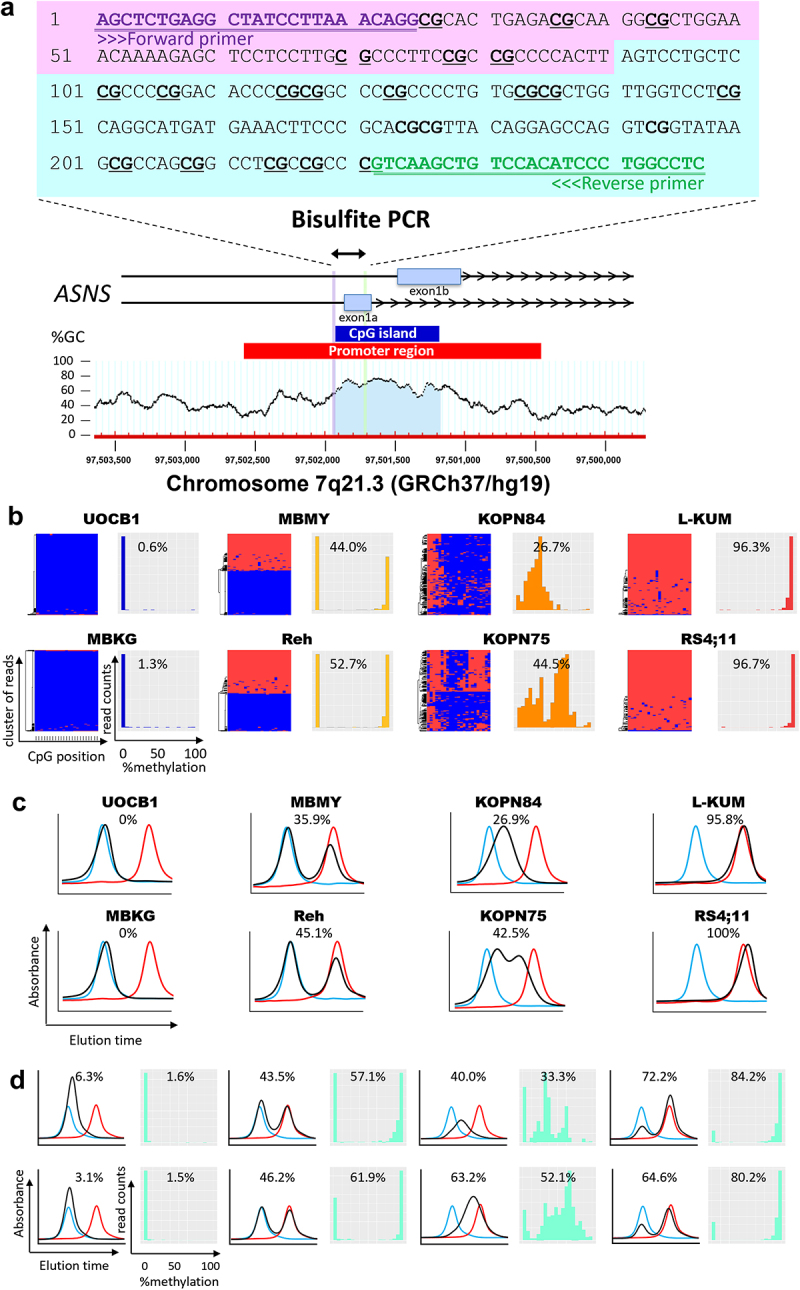
Reliability of HPLC chromatogram to evaluate the *ASNS* gene methylation status in BCP-ALL cell lines and clinical samples. (a) Schematic representation of a CpG island at the boundary between the promoter region and the initial exon of the *ASNS* gene. Bisulfite PCR was performed in the indicated 247 bp region containing 23 CpG sites. (b) Allele-specific methylation pattern of the *ASNS* gene in eight representative BCP-ALL cell lines with different methylation status. In each cell line, the left panel indicates the hierarchical clustering heatmap, while the right panel indicates the histogram based on each read of the NGS analysis. In the heatmap, red and blue indicate methylated and unmethylated CpG sites, respectively. In the histogram, percent value represents mean percent *ASNS* methylation of each NGS read. (c) HPLC chromatograms of eight representative BCP-ALL cell lines with different methylation status. The vertical axis indicates absorbance at 260 nm and the horizontal axis indicates the elution time. The black line indicates the elution curve of each sample, while the red and blue lines indicate the elution curves of fully methylated and fully unmethylated synthesized DNA fragments, respectively. Percentage value represents mean percent *ASNS* methylation based on the measurements in elution curve. (d) HPLC chromatogram of eight representative BCP-ALL clinical samples with different methylation status. In each sample, the left panel indicates the HPLC chromatogram, while the right panel indicates the histogram of the NGS read. Percentage values represent mean percent *ASNS* methylation evaluated with the HPLC method (left) and NGS method (right), respectively.

Using the residual bisulphite PCR products of the above NGS analysis, HPLC was performed as previously described [19]. Briefly, eluent-A was 25 mmol/L MES-NaOH buffer (pH 6.0), and eluent-B was the same buffer plus 2 mol/L guanidine sulphate. Bisulphite PCR products were separated on a gradient of 30–50% eluent-B for 10 min at a flow rate of 1.0 mL/min. The separated PCR products were detected at 260 nm. HPLC analysis was performed at a column temperature of 70°C using the LC-20A system (Shimadzu Corp., Kyoto, Japan) equipped with a stainless-steel column (20 × 4.6 mm I.D.) filled with the anion-exchange packing materials with electrostatic and hydrophobic properties. Calibration curves of fully unmethylated and fully methylated status were obtained from synthesized DNA fragments that were identical to 247 base pairs (contained 23 CpG sites) of the genomic sequence at the boundary region of the *ASNS* gene (Figure 1a), and their chromatograms were generated for fully unmethylated and fully methylated controls as well as for each sample. In each chromatogram, we measured three heights: (a) fully methylated peak, (b) intersection between fully methylated and fully unmethylated peaks, and (c) fully unmethylated peak (Supplemental Figure 1). Based on these measurements, we calculated the mean percent of *ASNS* methylation as follows: (a + 0.5b)/(a+b+c)×100.

Precise analyses of asparaginase sensitivities and *ASNS* gene and protein expression levels were done as previously reported [17]. Spearman’s rank correlation analysis and Kruskal–Wallis test in combination with Steel-Dwass *post hoc* test were done using R ver.4.1.1 software.

## Results

We evaluated the methylation status of 23 CpG sites at the boundary region of promoter and exon 1 of the *ASNS* gene (Figure 1a) using the HPLC method. Prior to the extensive analyses, we sought to confirm the rationality of the global methylation status of 23 CpG sites as a biomarker. We evaluated the association of asparaginase sensitivities (IC_50_ value) with each methylation status of 23 CpG sites in the *ASNS* promoter of 79 BCP-ALL cell lines, which were evaluated by the NGS method in our previous study [17]. As indicated in Supplemental Figure 2, Spearman’s correlation values (R^2^) in 23 CpG sites varied from 0.18 to 0.24 (median, 0.21), while Spearman’s value of mean methylation of 23 CpG sites was 0.21. Thus, although there were some variations, the validity of mean methylation status of 23 CpG sites was almost similar to that of each CpG site at least in association with asparaginase sensitivities. Accordingly, it would be feasible to use the global methylation status of 23 CpG sites as a biomarker.

We investigated the bisulphate PCR products of both 79 BCP-ALL cell lines and 63 BCP-ALL clinical samples, in which the precise methylation status was already evaluated by the NGS method as we previously reported [17]. First, we qualitatively investigated methylation patterns in eight BCP-ALL cell lines with different methylation status of the *ASNS* gene (Figures 1b-c). In UOCB1 and MBKG, histograms based on each read of NGS analysis revealed a sharp single peak of fully methylated reads (Figure 1b). The HPLC chromatogram consistently showed a single-peak, which was almost completely overlapped with the control elution curve of the fully methylated synthesized DNA fragments (Figure 1c). Next, in L-KUM and RS4;11 cell lines, NGS histograms revealed a sharp single-peak of fully unmethylated reads (Figure 1b). Consistently, the HPLC chromatogram clearly showed a single-peak, which was completely overlapped with the control elution curve of fully unmethylated synthesized DNA fragments (Figure 1c).

**Figure 2.**
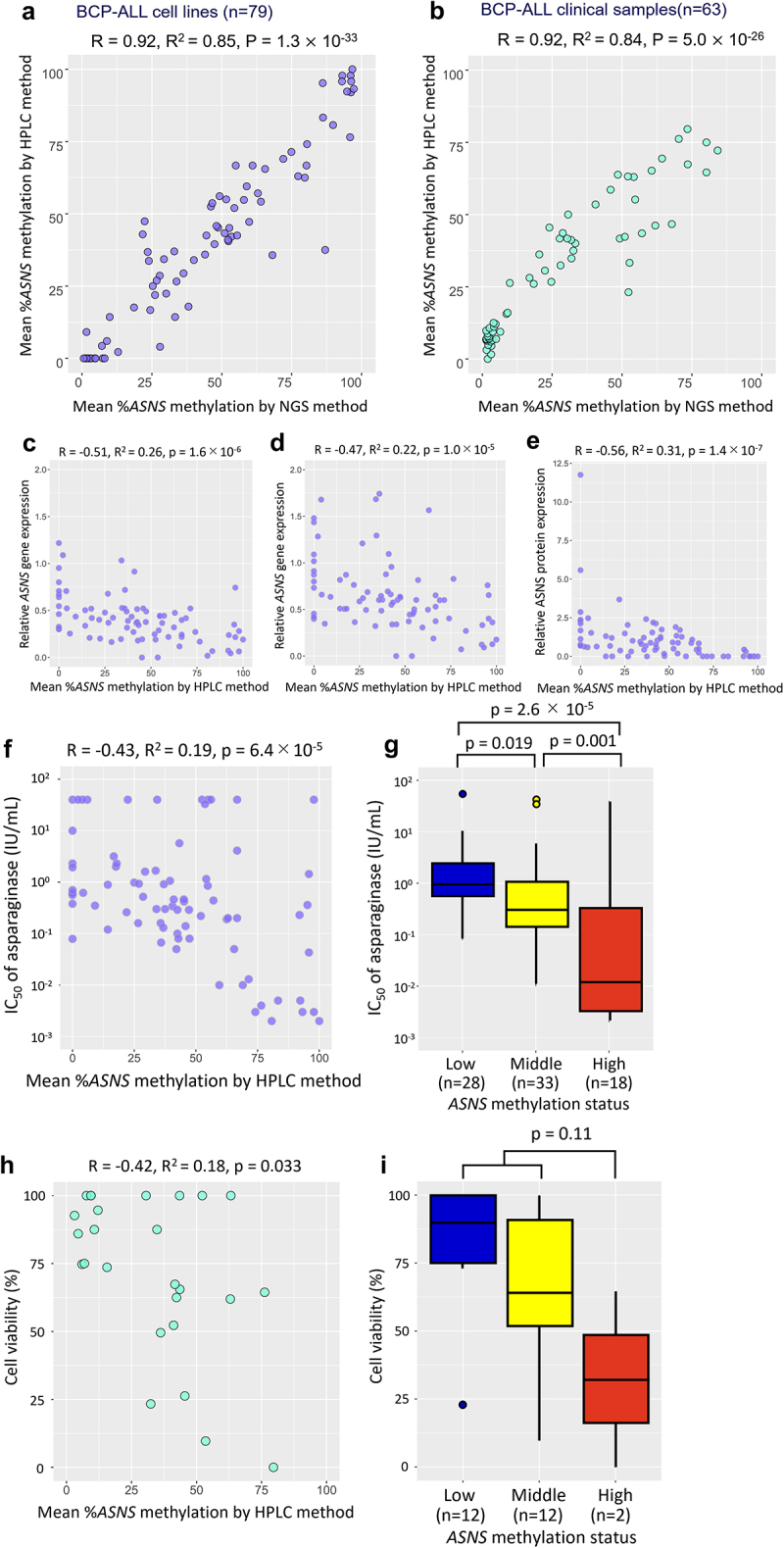
Utility of the *ASNS* methylation status evaluated with the HPLC chromatogram as a pharmacogenomic biomarker to predict asparaginase sensitivities of BCP-ALL cell lines and clinical samples. (a,b) Correlation of mean percent *ASNS* methylation evaluated by the HPLC method (vertical axis) with that by NGS method (horizontal axis) in 79 BCP-ALL cell lines (a) and 63 BCP-ALL clinical samples. (b) Correlation coefficients and p-values in Spearman’s rank correlation coefficient are indicated at the top of the panels. (c,d,e) correlations of mean percent *ASNS* methylation evaluated by the HPLC method with basal (c) and asparaginase-induced (d) *ASNS* gene expression levels, and ASNS protein expression levels (e). (f,g) correlations of mean percent *ASNS* methylation evaluated by the HPLC method (f) and association of *ASNS* gene methylation status (g) with IC_50_ values of asparaginase in 79 BCP-ALL cell lines. *P* values in Steel–Dwass *post hoc* test for Kruskal–Wallis test are indicated on the top. (h,i) correlation of mean percent *ASNS* methylation (h) and association of *ASNS* gene methylation status (i) evaluated by the HPLC method with *in vitro* cell viabilities treated with 0.01 IU/ml of asparaginase for 72 hours in 26 BCP-ALL clinical samples at diagnosis. *P* values in Mann-Whitney U test are indicated at the top of the figure.

Next, we focused on moderately methylated cell lines. In MBMY and Reh, NGS histograms revealed two sharp peaks of fully methylated and fully unmethylated reads corresponding to mono-allelic methylation (Figure 1b). The HPLC chromatogram showed two peaks, which were overlapped with control elution curves of both fully methylated and unmethylated synthesized DNA fragments (Figure 1c). Moreover, in KOPN84, the NGS histogram revealed a relatively wide single peak of partly methylated reads (Figure 1b). Consistently, HPLC chromatograms confirmed a single wide peak of weak methylation (Figure 1c). Furthermore, in KOPN75, NGS histograms revealed two peaks of partly unmethylated and partly methylated reads (Figure 1b). Consistently, the HPLC method showed two peaks of almost unmethylated and moderately methylated status (Figure 1c). Overall, the patterns of the HPLC chromatograms in the 79 BCP-ALL cell lines were identical to those of the NGS histograms (Supplemental Figure S3). Similarly, as representatively shown in eight clinical samples with different methylation status (Figure 1d), patterns of the HPLC chromatogram in 63 BCP-ALL clinical samples were identical to those of the NGS histogram (Supplemental Figure S4). Thus, we quantified the mean percent methylation of the *ASNS* gene using indicated formula (Supplemental Figure 1). In 79 BCP-ALL cell lines, a strong positive correlation was confirmed between mean percent methylation evaluated with the HPLC method and that with the NGS method (R^2^ = 0.85, *p* = 1.3 × 10^−33^; Figure 2a). Moreover, a strong positive correlation was also observed in 63 BCP-ALL clinical samples (R^2^ = 0.84, *p* = 5.0 × 10^−26^; Figure 2b). These observations demonstrated that the HPLC chromatogram of bisulphate PCR product is a reliable method to qualitatively and quantitatively evaluate methylation status of the *ASNS* gene.

Next, we evaluated the pharmacogenomic utilities of the *ASNS* gene methylation status evaluated with the HPLC analysis. As we previously observed in the *ASNS* gene methylation status evaluated with the NGS analysis [17], both basal (R^2^ = 0.26, *p* = 1.6 × 10^−6^; Figure 2c) and asparaginase-induced (R^2^ = 0.22, *p* = 1.0 × 10^−5^; Figure 2d) *ASNS* gene expression levels in 79 BCP-ALL cell lines were significantly correlated with mean percent methylation evaluated with the HPLC method. Moreover, the basal ASNS protein expression levels of 79 BCP-ALL cell lines were significantly correlated with mean percent methylation evaluated with the HPLC method (R^2^ = 0.31, *p* = 1.4 × 10^−7^; Figure 2e).

Indeed, the IC_50_ values in asparaginase sensitivities were marginally correlated (R^2^ = 0.19, *p* = 6.4 × 10^−5^; Figure 2f) with mean percent methylation evaluated with the HPLC method. Of note, the IC_50_ values in 18 highly (≥66.7%) methylated cell lines (median IC_50_, 0.012 IU/mL) were significantly lower than those in the 33 moderately (33.4–66.6%) methylated cell lines (0.30 IU/mL, *p* = 1.0 × 10^−3^) and the 28 weakly (≤33.3%) methylated cell lines (0.91 IU/mL, *p* = 0.019) (Figure 2g).

Based on these observations, we finally compared the utilities of % methylation values evaluated by the HPLC method with those by the NGS method [17] in terms of correlations with *ASNS* gene and protein expression levels as well as IC_50_ values (Supplemental Figure S5). Each value of correlation coefficient (R) was as follows: basal *ASNS* gene expression level, −0.51 (HPLC) vs −0.46 (NGS); asparaginase-induced *ASNS* gene expression level, −0.47 (HPLC) vs −0.40 (NGS); basal ASNS protein expression level, −0.56 (HPLC) vs −0.57 (NGS); and IC_50_ for asparaginase, −0.43 (HPLC) vs −0.46 (NGS). Thus, in terms of these correlations, although their superiority was not confirmed, % methylation values evaluated by the HPLC method were at least as reliable as those by the NGS method.

Additionally, we evaluated the significance of *ASNS* methylation status evaluated with the HPLC method in clinical samples. We utilized cell viabilities of 26 BCP-ALL samples at diagnosis treated with 0.01 IU/mL of asparaginase *in vitro* for 72 h, which were previously reported [17]. The asparaginase sensitivities were marginally correlated with mean percent methylation evaluated with the HPLC method (R^2^ = 0.18, *p* = 0.033; Figure 2h). Although the sample size was limited, cell viabilities in two highly methylated clinical samples (median; 32%) were relatively lower than those in 12 moderately methylated and 12 weakly methylated clinical samples (81%, *p* = 0.11) (Figure 2i). These observations indicated that mean percent methylation of the *ASNS* gene evaluated with the HPLC method is a useful pharmacogenomic biomarker to predict asparaginase sensitivity in BCP-ALL specimens.

## Discussion

NGS analysis is a reliable and powerful technology for evaluating the methylation status of multiple regions at high resolution in a large number of specimens [20]. However, although costs for NGS amplicon sequencing are steadily decreasing, NGS analysis is still costly when applied for a few clinical samples and time-consuming in the evaluation of specific bisulphite PCR products [21]. In these aspects, although the initial investment in HPLC equipment is significant, HPLC is relatively cost-effective due to its high accuracy and precision across individual runs with capabilities for analysing multiple samples in a short amount of time. In fact, HPLC has been widely employed for laboratory tests [22] and drug monitoring [23]. Moreover, we recently reported [24] that the HPLC method was a powerful tool in glioblastoma cases to evaluate the methylation status of the *MGMT* gene, which is a well-known biomarker to predict the clinical response to temozolomide in glioblastoma cases [25,26]. In this study, we validated that both pattern and mean percent methylation of the *ASNS* gene evaluated with the HPLC method of bisulphate PCR products in BCP-ALL cell lines and clinical samples were identical to those evaluated with our previous NGS study [17]. Moreover, mean percent methylation of the *ASNS* gene evaluated with the HPLC method was correlated with levels of basal and asparaginase-induced *ASNS* gene expression and basal ASNS protein expression in BCP-ALL cell lines. Of clinical importance, it was also associated with *in vitro* asparaginase sensitivities of both BCP-ALL cell lines and clinical samples at diagnosis. These observations indicated that the *ASNS* gene methylation status evaluated with the HPLC method is a pharmacogenomically reliable biomarker for predicting the asparaginase sensitivity of BCP-ALL.

In our previous study, we investigated the association of chromosomal abnormalities with *ASNS* gene methylation in three large clinical cohorts of childhood BCP-ALL by evaluating mean percent methylation of six CG dinucleotides located in the CpG islands of the *ASNS* gene [17]. In all three cohorts, highly methylated status corresponding to the bi-allelic methylation was most frequently observed in BCP-ALL with favourable karyotypes such as high-hyperdiploid and *ETV6::RUNX1*, whereas it was rarely observed in BCP-ALL with unfavourable karyotypes such as *MLL* rearrangement and *BCR::ABL1* [17]. In high-hyperdiploid BCP-ALL cases, simultaneous trisomy of chromosomes 4, 10, and 17, which is particularly associated with a good prognosis, showed the highest level of *ASNS* methylation [17].

These findings indicated that higher asparaginase sensitivity due to higher *ASNS* methylation status is one of the backgrounds for better outcomes in childhood BCP-ALL with favourable karyotypes. Moreover, in intermediate-risk karyotypes, *ASNS* methylation levels in iAMP21 cases were almost as high as those in favourable karyotypes [17]. Of note, event-free survival of iAMP21 patients was dramatically improved in the treatment regimen with intensified asparaginase therapy [27]. In this context, it is noteworthy that approximately two-thirds of childhood BCP-ALL with favourable karyotypes showed weakly or intermediately methylated status of the *ASNS* gene [17].

Accordingly, there are two possible utilities of HPLC-based *ASNS* methylation status for precision medicine in childhood BCP-ALL. One is that highly methylated cases with favourable karyotypes might be candidates for treatment regimen with reduced intensity of chemotherapeutic agents other than asparaginase. In this group, favourable outcomes might be assured by higher asparaginase sensitivity owing to bi-allelic methylation of the *ASNS* gene, even in the treatment regimen with reduced intensities of other chemotherapeutic agents. The other utility is that weak or intermediately methylated cases with favourable karyotypes might be candidates for intensified treatment regimen with chemotherapeutic agents other than asparaginase. In this group, based on lower asparaginase sensitivity due to mono-allelic or bi-allelic unmethylation of the *ASNS* gene, therapeutic outcome would be further improved by the treatment regimen in which other chemotherapeutic agents are intensified. In the NOPHO database [17], among 163 *ETV6::RUNX1*-positive and 187 hyperdiploid cases, the *ASNS* gene was weakly methylated in 31 (19%) and 107 (57%) cases, respectively. Thus, approximately one-third of the cases with these favourable karyotypes were thought to be relatively resistant to asparaginase due to weak methylation of the *ASNS* gene, and subsequently, their prognosis could be further improved by the intensified treatment regimen with chemotherapeutic agents other than asparaginase.

Similar utility is assumed in T-ALL patients, since the prognosis of the patients with highly methylated status of the *ASNS* gene was significantly superior to that of the patients with weakly methylated status [18,28]. Considering the adverse effects of asparaginase [11,12], although intensified asparaginase therapy improved the therapeutic outcome of T-ALL [28], further intensification of asparaginase therapy might not be recommended in T-ALL patients with intermediately or weakly methylated status of the *ASNS* gene. Alternatively, in this group of patients, intensification of chemotherapeutic agents other than asparaginase would be beneficial.

In the present study, there are several technical and application limitations. First, in the clinical specimen, our method could not differentiate contaminated normal haematopoietic cells from leukaemia cells. Since the *ASNS* gene is basically unmethylated in normal haematopoietic cells [17], contamination of normal haematopoietic cells could underestimate the *ASNS* gene methylation status of leukaemic samples. Second, our method is not technically applicable to the genomic samples that had been previously expanded using PCR-based amplification. Third, variation in the IC_50_ values of asparaginase in the intermediately methylated samples was quite large compared to those in the highly and weakly methylated samples. Thus, the utility of *ASNS* gene methylation status in clinical practice could be relatively limited to certain highly methylated and weakly methylated cases, which could correspond to good and poor asparaginase sensitivities, respectively. Fourth, although the *ASNS* methylation status of ALL cells is significantly associated with asparaginase sensitivity *in vitro*, its utility as a biomarker for asparaginase sensitivity in clinical practice has not yet been validated. Thus, possible use of *ASNS* gene methylation status as a biomarker for a risk-stratification approach in the treatment for childhood ALL must be validated in further prospective studies.

In summary, to implement the above precision medicine, evaluation of methylation status of the *ASNS* gene is clinically crucial at the earliest stage of diagnosis. Considering its convenience, HPLC analysis of the *ASNS* gene methylation status would be useful in precision medicine for asparaginase therapy.

## Supplementary Material

Supplemental Material

## Data Availability

The datasets used and analysed during the current study are not publicly available, but inquiries can be made to the corresponding author.
