# Supplementary material for: Utility of ASNS gene methylation evaluated with the HPLC method as a pharmacogenomic biomarker to predict asparaginase sensitivity in BCP-ALL
Source: Epigenetics. 2023 Oct 15;18(1):2268814. doi: 10.1080/15592294.2023.2268814 (PMC10578186; doi:10.1080/15592294.2023.2268814)

**Supplemental Figure 1.** Quantification of mean percent *ASNS* methylation based on the HPLC chromatogram. Mean percent *ASNS* methylation was quantified as (a+0.5b)/(a+b+c)×100, based on the following measurements: (a) height of fully methylated peak corresponding to the peak of red line, (b) height of intersection between fully methylated and fully unmethylated peaks, and (c) height of fully unmethylated peak corresponding to the peak of blue line.

**Supplemental Figure 2.** Correlation between *in vitro* asparaginase sensitivity (IC_50_) and methylation rate of 23 CpG sites at the promoter and exon1 boundary region of the *ASNS* gene in 79 BCP-ALL cell lines. The methylation rate of each CpG site was evaluated by the NGS method in our previous study [18]. The color scale in the first column indicates IC_50_ value of each cell line (from the most sensitive at the top to the most resistant at the bottom), while that in each row indicates % methylation of each CpG site. Spearman’s correlation values (R^2^) between IC_50_ values of asparaginase and methylation rates of each CpG sites are indicated at the top of the panel.

**Supplemental Figure 3.** Comparison of the HPLC chromatogram (left) and the NGS histogram (right) in 79 BCP-ALL cell lines. In each sample, the left panel indicates the HPLC chromogram, while the right panel indicates the histogram of each NGS read. Mean percent *ASNS* methylation evaluated with the HPLC method (left) and NGS method (right) are indicated at the top of each panel.

**Supplemental Figure 4.** Comparison of the HPLC chromatogram (left) and the NGS histogram (right) in 63 BCP-ALL clinical samples. In each sample, the left panel indicates the HPLC chromogram, while the right panel indicates the histogram of each NGS read. Mean percent *ASNS* methylation evaluated with the HPLC method (left) and NGS method (right) are indicated at the top of each panel.

**Supplemental Figure 5.** The relationship between *ASNS* methylation status evaluated using NGS method and *ASNS* gene expression, ASNS protein expression, and asparaginase sensitivities in BCP-ALL cell lines. **(A,B,C)** Correlations of mean percent *ASNS* methylation evaluated by the NGS method with basal **(A)** and asparaginase-induced **(B)** *ASNS* gene expression levels, and ASNS protein expression levels **(C)**. **(D,E)** Correlations of mean percent *ASNS* methylation evaluated by the NGS method **(D)** and association of *ASNS* gene methylation status **(E)** with IC_50_ values of asparaginase in 79 BCP-ALL cell lines. P values in Steel-Dwass *post hoc* test for Kruskal-Wallis test are indicated on the top.


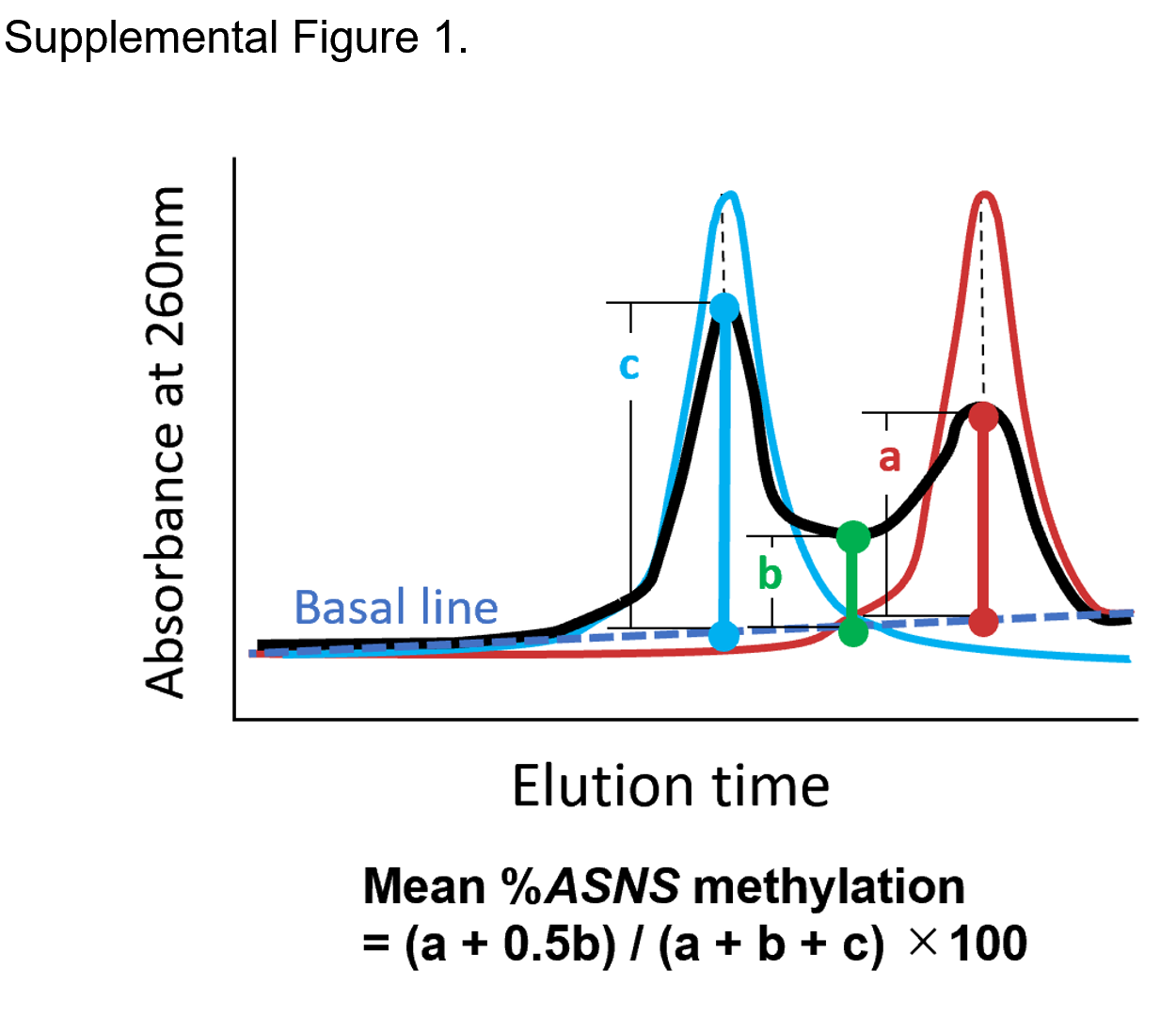


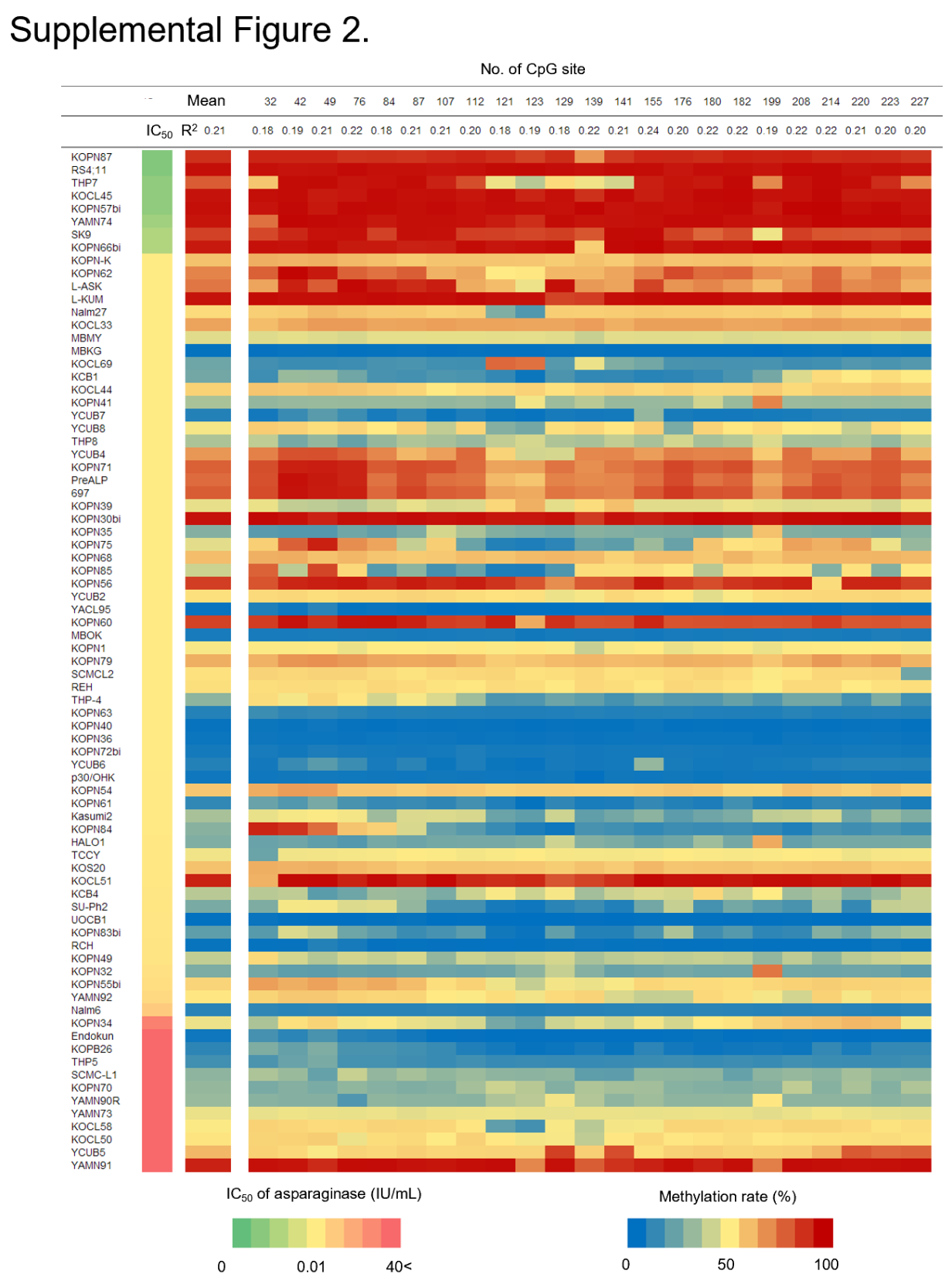


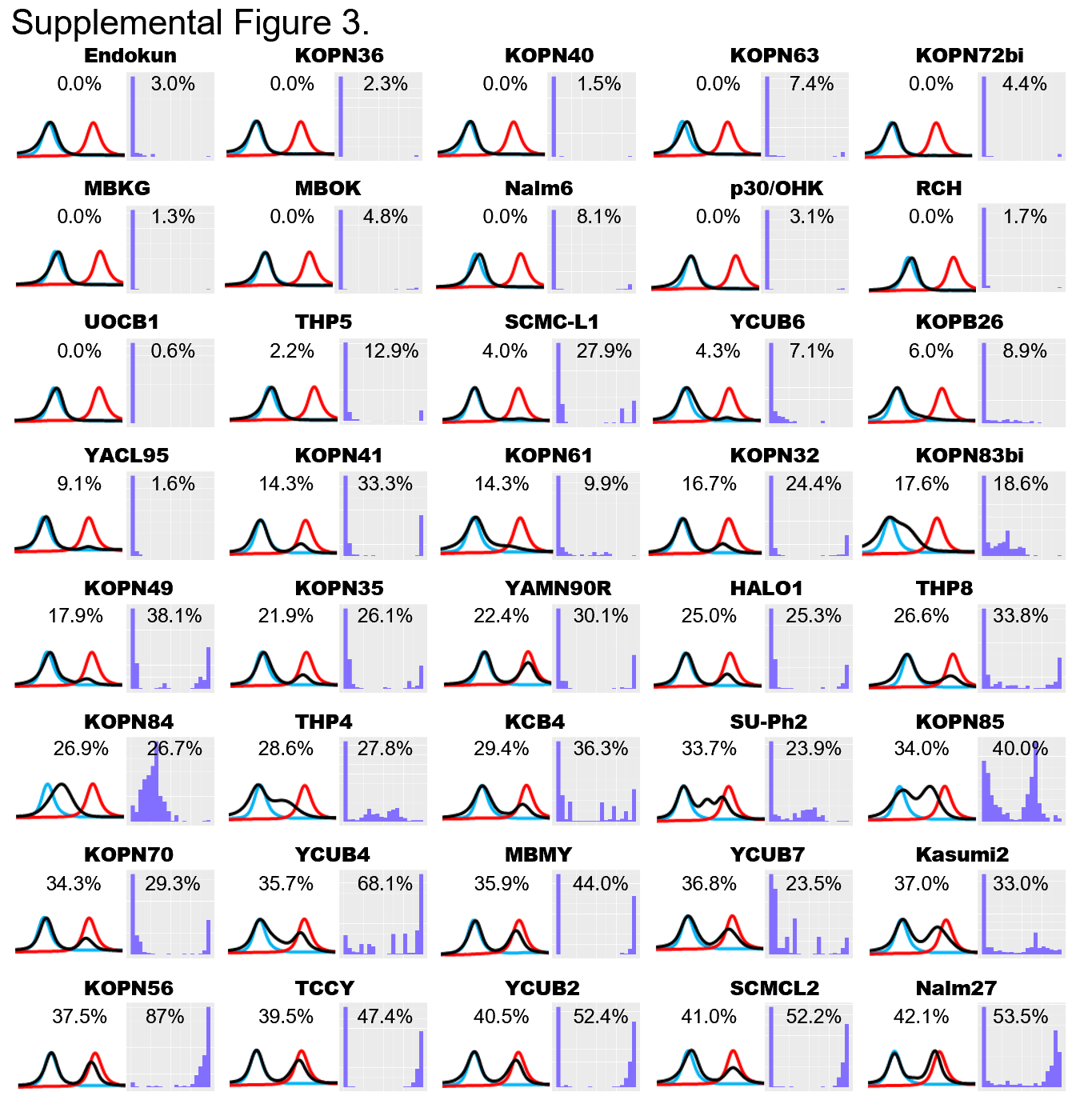


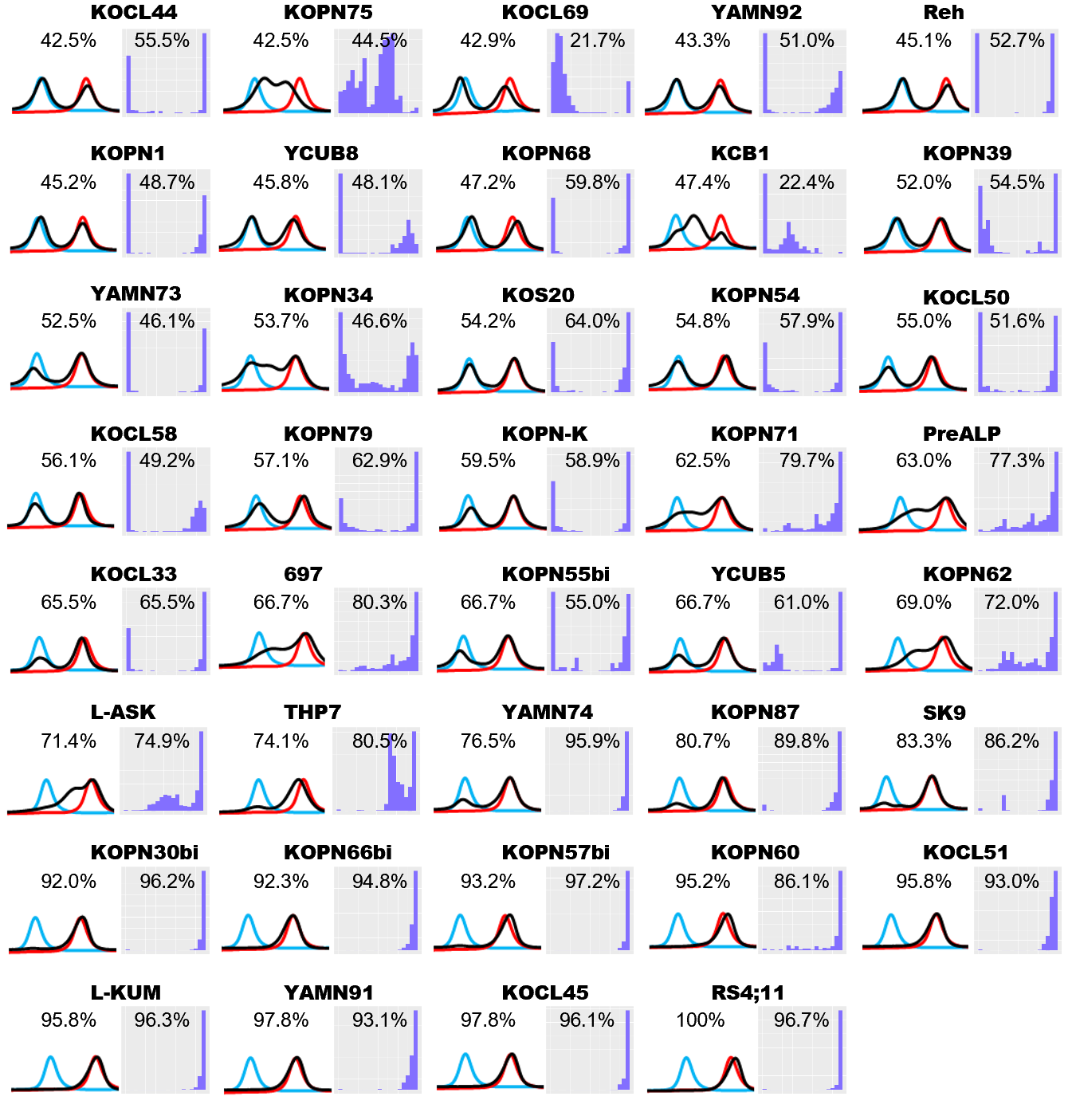


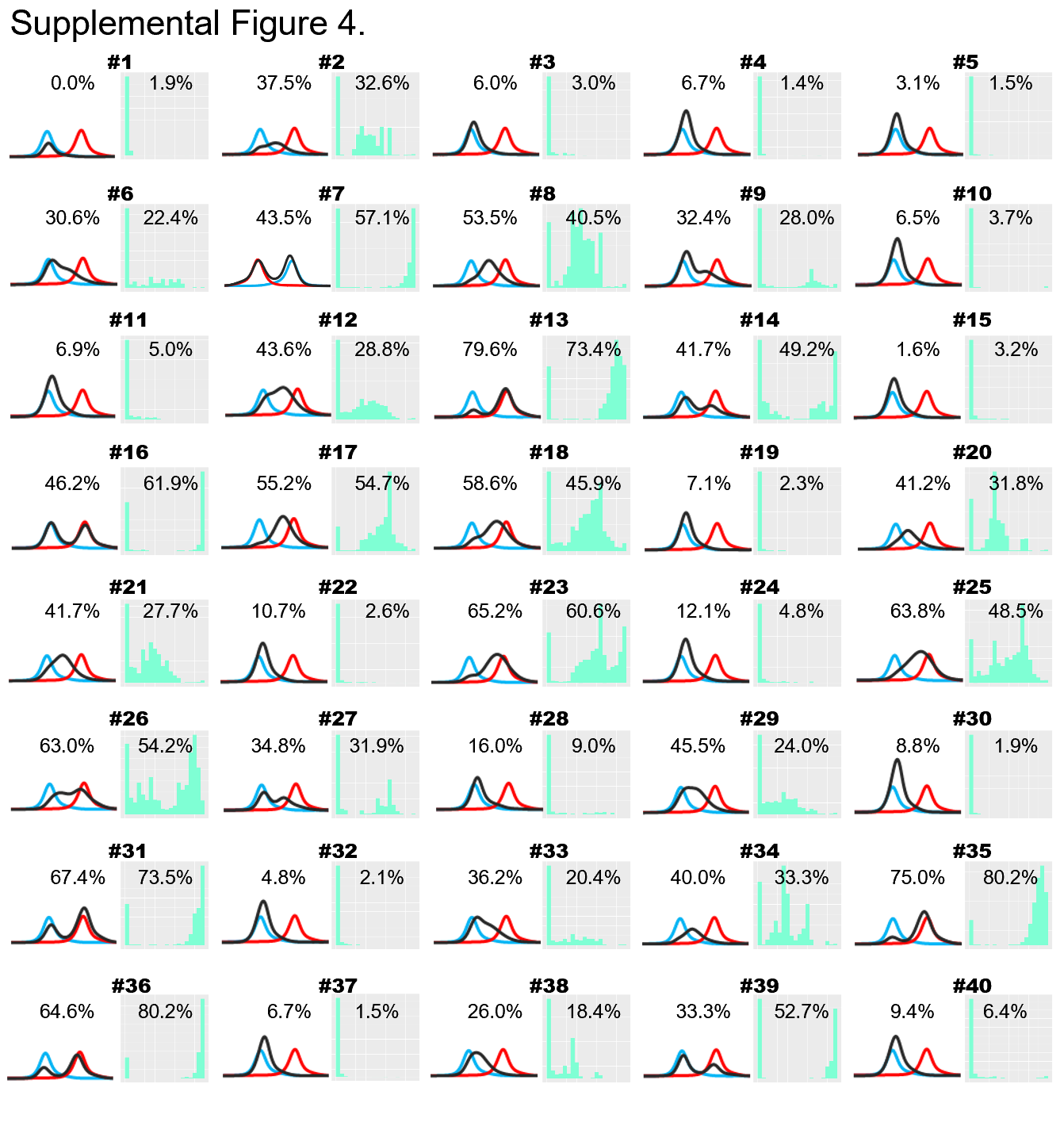


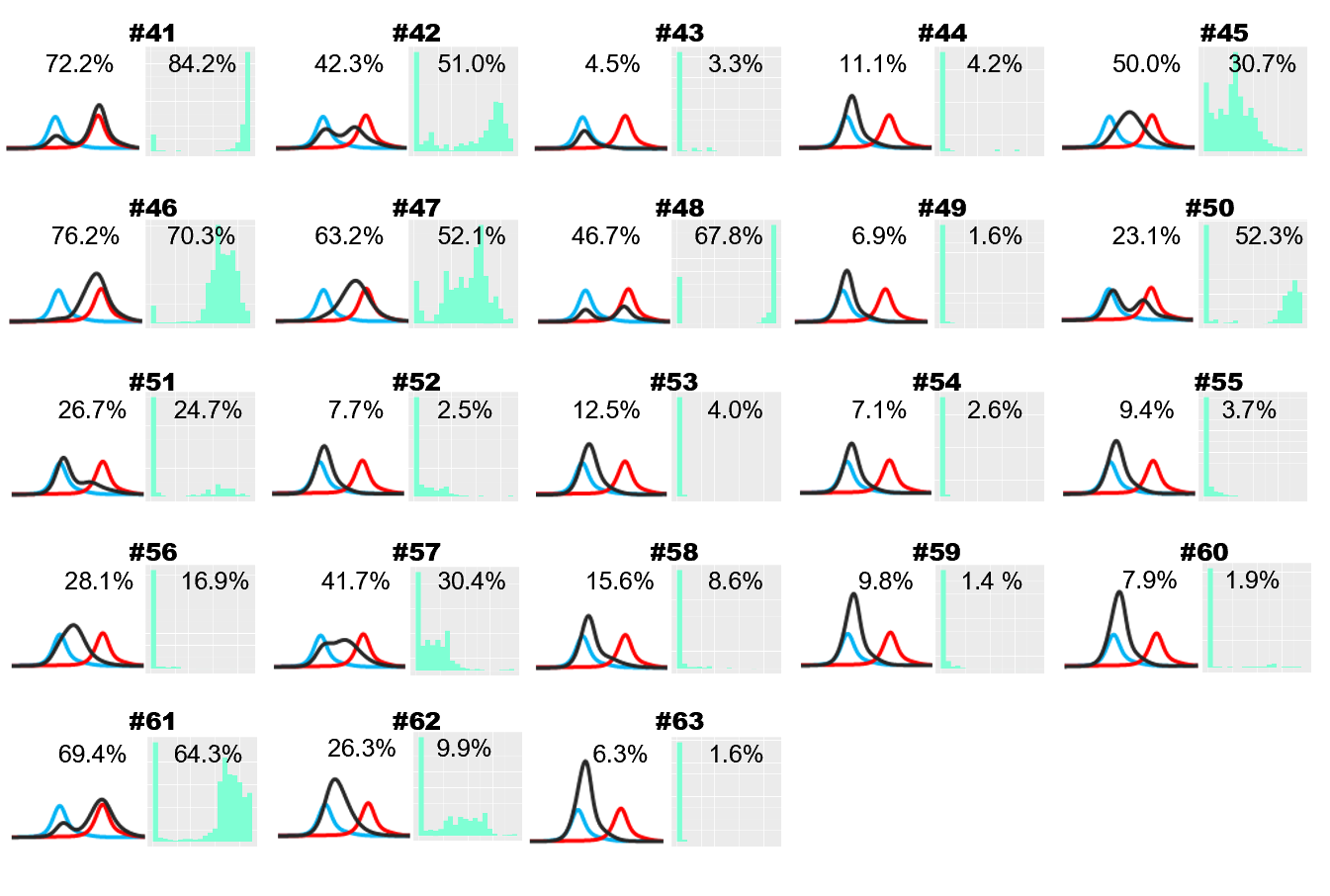


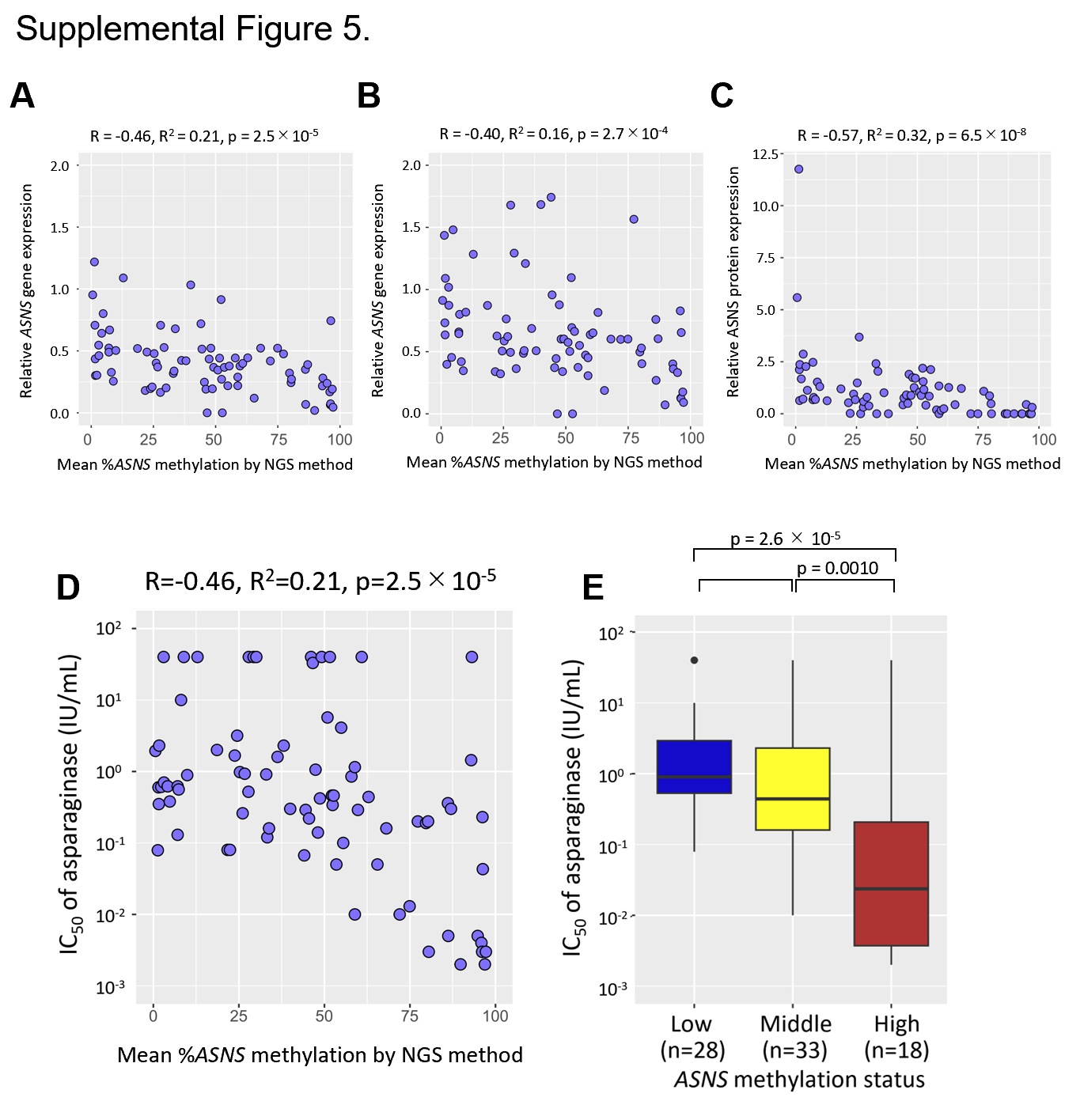

Supplement: Supplemental Material [file KEPI_A_2268814_SM7644.docx]
